# Characterization of Simple and Double Yeast Cells Using Dielectrophoretic Force Measurement

**DOI:** 10.3390/s19173813

**Published:** 2019-09-03

**Authors:** Fernando-Juan García-Diego, Mario Rubio-Chavarría, Pedro Beltrán, Francisco J. Espinós

**Affiliations:** 1 ACUMA Research Center Universitat Politècnica de València, Av. de los Naranjos s/n, 46022 Valencia, Spain; 2 Escuela Técnica Superior de Ingenieros Industriales Universitat Politècnica de València, Av. de los Naranjos s/n, 46022 Valencia, Spain

**Keywords:** yeast cell, simple, double, DEP, dielectrophoresis, measurement, stokes

## Abstract

Dielectrophoretic force is an electric force experienced by particles subjected to non-uniform electric fields. In recent years, plenty of dielectrophoretic force (DEP) applications have been developed. Most of these works have been centered on particle positioning and manipulation. DEP particle characterization has been left in the background. Likewise, these characterizations have studied the electric properties of particles from a qualitative point of view. This article focuses on the quantitative measurement of cells’ dielectric force, specifically yeast cells. The measures are obtained as the results of a theoretical model and an instrumental method, both of which are developed and described in the present article, based on a dielectrophoretic chamber made of two V-shaped placed electrodes. In this study, 845 cells were measured. For each one, six speeds were taken at different points in its trajectory. Furthermore, the chamber design is repeatable, and this was the first time that measurements of dielectrophoretic force and cell velocity for double yeast cells were accomplished. To validate the results obtained in the present research, the results have been compared with the dielectric properties of yeast cells collected in the pre-existing literature.

## 1. Introduction

Despite the huge increase of dielectrophoretic force (DEP) related technologies, developed in the last twenty years [1], there is still a great deal of applications to be studied. During this time, several revolutions have taken place regarding the use of DEP and dielectrophoretic devices. After key contributions in material sciences, electronic engineering, and nanoscience, today DEP is being focused on new applications in chemical and biochemical analysis [1]. 

One of the points whereby DEP is being considered a potential tool for chemical and biochemical analysis is its usefulness for micromanipulation [2,3,4,5,6,7]. Among all the possible applications, especially relevant are those related to microparticle detection, positioning, or stratification [8,9,10,11,12,13], and to a lesser extent, the characterization applications [14,15,16,17]. The main two advantages of DEP against other characterization and/or manipulation techniques are: I) DEP techniques present high sensitivity and specificity [18,19,20,21,22,23] and II) they are cheap and easy to use [14]. Regarding the microparticle characterization, DEP employment, as one of the bases of "label-free" techniques, is especially useful [14,24,25,26,27]. Another important line of research on dielectrophoresis is to attempt to integrate on the same device the microfluidic channels for cell manipulation and all the laboratory functions (hardware and software). This is the so-called lab-on-chip [18,24,28,29,30,31].

With regard to the DEP, the latest publications on the study of microparticle characterization focus on: cellular capture and stratification [28], cancer cell characterization [24,25,26], viral detection and characterization [5,9,23,29], DNA detection [32,33,34], and bacterial identification and detection [15,35].

Recently, methods capable of achieving cellular characterization from the qualitative measurement of the alteration of the cell’s mechanical properties have been developed. Conversely, there are few works focused on the measurement of the dielectric parameters that enable us to obtain the value of the exerted DEP over the cells [16,17,36,37,38]. These research articles, as well as this one, based their theoretical models on the mathematical expression of DEP given by Pohl [39].

Working with DEP characterization presents two main problems. The first of them is the Joule effect by means of creation of convection currents; these currents are able to overshadow the action of DEP. Likewise, the Joule effect combined with the electric field is capable of damaging the studied cells [40]. Most of the time, the Joule effect could be avoided by using high frequencies and non-conductive solutions. The second problem is that the electrode geometry, particle position [41], conductivities, and permeabilities of particle and medium should be known. However, with the proposed experimental model, none of these problems is seen in the frequency spectrum employed. Moreover, the model, geometry, and position of the particles are well known, because we have a 2D electric field that could be analyzed by an optic microscope.

This article presents a theoretical model and an instrumental method as tools for the characterization of DEP exerted over microparticles by means of an imaging process. Applying Newton’s Second Law and Stokes’ frictional force, through the measurement of speed, it will be possible to characterize the exerted DEP over microparticles, concretely *Saccharomyces cerevisiae* cells (yeast cells). The importance of this work is rooted in its applications in the field of cellular characterization, being one of the few whose results about the exerted DEP over the particles are measurable and repeatable.

In the previous research, it has been possible to quantify the DEP exerted over yeast cells of the same dielectric features of the membrane [16], and the ability to characterize the population radius has been achieved.

**Figure 1:**
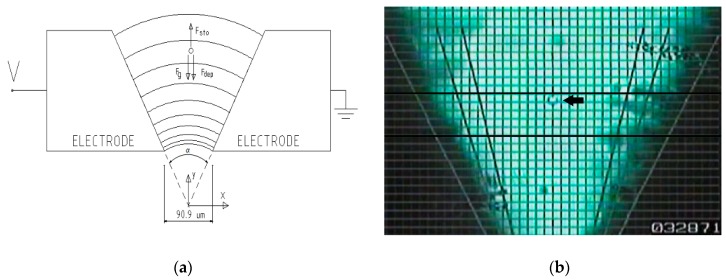
(**a**) Chamber and dynamic model diagram. Afterwards, α is defined as the angle between electrodes and β the angle between the electrode and the *X*-axis. Both are referred to in this figure. (**b**) Experiment frame. You can see one single measured cell (arrow) and the pearl chains following the electric field lines. The two thickest parallel lines where the measurements were made are in the lower right corner the frame counter.

The method used in this work is based on the measure of the cellular velocity and the calculation of the DEP using the Stokes dynamic model. The chamber, Figure 1a, is made of two electrodes placed following the shape of a “V”. This is the way in which a non-uniform electric field is obtained. In previous works [16,17], this model has already been applied to single cells to study its dimensions. This article is the first that exposes its application to the coupled yeast cells and studies its dielectric characteristics.

## 2. Theoretical Model

The DEP is obtained when a particle is introduced in a non-uniform electric field and media of different polarizability. This effect is created because of the dipole interaction with the field gradient [39]. The DEP force ($F_{DEP}$) acting over a particle (considered a single sphere) depends on its volume (ν), on the effective permittivity of medium-particle ($\varepsilon_{mp}$), and on the square of the electric field gradient intensity $\nabla E^{2}$:

$$F_{DEP}=\frac{3}{2}\nu \varepsilon_{mp}\nabla E^{2}$$

The εmp=εmRe[fCM], where $\varepsilon_{m}\;$is the permittivity of the medium and Re[f_CM_] is the real component of the Clausius–Mossotti factor [39]. 

This equation of the DEP force and the values it can take is widely discussed in the literature [42,43,44,45,46,47].

In our experiment, a chamber of two “V” shaped electrodes is used (Figure 1a). In this figure, the forces acting on a particle in the axis of symmetry of the chamber are also schematized. A frame of the experiment can be seen in Figure 1b, and particles that descend along the axis of symmetry of the chamber and the "pearl chains" that are oriented according to the electric field lines can be seen.

With this setup, a bi-dimensional electric field is obtained, having the advantages of the formulation of the electric field and that the cellular movement is developed in parallel planes, which permits its observation through the two dimensional image of a microscope. 

The cellular trajectories are located in the XY plane; only cells with vertical trajectories (*Y*-axis) were chosen for the measurements, as shown in Figure 1a. These trajectories are in symmetry with the axis of the preparation, so the electrodes’ proximity effect is well-adjusted [16]. 

Following Newtons’ Second Law, the cells are found in balance, so:

$$F_{g}+F_{DEP}+F_{sto}=m\cdot a$$

Because of its small value, the mass by acceleration term is considered negligible in relation to the other force values [16,17]:

(1) $$F_{g}+F_{DEP}+F_{sto}=0$$

$F_{g}$ is the effective weight of the particle in the medium

(2) $$F_{g}=\nu \rho_{pm}g$$

where $\rho_{pm}$ is the difference between the particle’s and medium’s density and g is the acceleration of gravity.

### 2.1. Linear Relationship between Velocity and Position of a Single Cell

$F_{DEP}$ is the DEP force that is expressed in terms of the difference between the electrode’s potential in root mean square (RMS) volts (*V*) and $F\left(\vec{r}\right)$, the position in the form of a vectorial function [44].

$$F_{DEP}=\frac{3}{2}\nu \varepsilon_{mp}V^{2}F\left(\vec{r}\right)$$

$F_{sto}$ is the Stokes’ viscous friction force that opposes the movement; its expression for a single spherical cell is [16]:

$$F_{sto}=-6\pi \eta Rv_{p}$$

where *η* is the medium’s dynamic viscosity, R denotes the cellular radius, and $v_{p}$ represents the particle velocity. 

The Reynolds number $N_{R}$, for the case of a spherical solid of radius R in movement immersed in a liquid, is defined by the following expression [48]:

$$N_{R}=\frac{\rho v_{p}R}{\eta }$$

in which ρ represents the fluid’s density. 

The Stokes’ law requires to a Reynolds number lower than 1, and a concentration percentage in a volume lower than 0.1% [49]. In our experiment, both conditions have been satisfied as discussed later.

Substituting all these forces and rearranging terms results in the following velocity expression [16] in the *Y*-axis (Figure 1a):

(3) $$v_{p}=\frac{R^{2}\varepsilon_{pm}V^{2}}{3\eta }F\left(\vec{r}\right)+v_{s}$$

where $v_{s}=\frac{2R^{2}\rho_{pm}g}{9\eta }$ is the sedimentation velocity of a single cell.

In our experiment $F\left(\vec{r}\right)$ takes the value of [16,17]

$$F\left(\vec{r}\right)=\frac{-2}{\alpha^{2}y^{3}}\vec{j}$$

If it is substituted in Equation (3), the module of the velocity of an only spherical cell falling along the *Y*-axis is obtained (Figure 1a,b) as follows [16]:

(4) vp=2R2εmRe[fCM]V23ηα2y3+vs

All velocities in Equation (4) are in $-\vec{j}$. Also, this equation indicates that the induced velocity by the DEP in a determined position is a function of the square of the cellular radius R^2^ and the effective polarizability Re[f_CM_]. The linear relation of Equation (4), velocity versus cellular position function y^−3^, allows us to verify the behavior of one single cell and to characterize it [16].

### 2.2. Linear Relationship Between Velocity and Position of a Coupled Cell

Analogously, for coupled cells:

(5) $$F_{sto}=-6\pi \eta fRv_{p}$$

where “fR” is the equivalent sphere radius. In the case of two spheres together whose movement is perpendicular to the axis, which bounds both centers, f takes the value of “1.44” [50]. The expression of the DEP in the case of two spherical particles is extracted from [51]:

(6) FDEP=4πR3εmα2y3Re[2fCM1−fCM4]V2(RMS)

If Equations (2), (5), and (6) are replaced in Equation (1), Equation (7) is obtained as follows:

(7) vp=23R2εmRe[2fCM1−fCM4]1.44ηα2V2y−3+vs

Being $v_{s}=\frac{4R^{2}\rho_{mp}g}{1.44\eta 9}$, the sedimentation velocity of two cells. 

### 2.3. Mathematical Relationship between Velocity and Position of a Single and Coupled Cell

In this way, it can be appreciated that Equations (4) and (7) are of the following form:

(8) $$v=F_{1}V^{2}y^{-3}+v_{s}$$

Being $F_{1}$ the value of the speed to one V (RMS) value $v_{s}$ and sedimentation rate. The value of each parameter is shown in Table 1.

Because of the electrode’s polarization, theoretical $F_{1}$ differs from the experimental one ($F_{1,EX}$). So, the correction factor *k*(*ω*) is applied as follows [16,52]:

(9) $$F_{1,EX}=k\left(\omega \right)F_{1}$$

Therefore, Equation (8) is presented in the experimental form as:

(10) $$v=F_{1,EX}V^{2}y^{-3}+v_{s}$$

## 3. Materials and Methods

### 3.1. Dielectrophoretic Device

The dielectrophoretic device was built from two silver and gold plated electrodes of 5 × 20 × 2 mm, that formed an angle of 53.13°, with a minimum separation of 90.9 μm between them. The vertical plane of the electrodes allows the cells to displace without scraping against the crystals that confine the solution and far from the electrode’s borders. To avoid heating the sample, optical fiber was used to light the microscope. A sinusoidal (30 V peak-to-peak) signal was applied AC Tektronix-CFG280 (Beaverton, Oregon, USA), which was capable of generating a frequency from 10 kHz to 10 MHz. The signal was monitored by means of a digital Tektronix TDS 320 (Beaverton, Oregon, USA) oscilloscope (100 MHz, 50 Ms/s). 

As described in other studies [16,52], a measure of the electric properties of the dielectrophoretic chamber was conducted to quantify the electrodes’ polarixation and find the *k*(*ω*) of Equation (9) of our experiment.

### 3.2. Cellular Suspension

As it is important that the largest possible number of cells are in the same physiological state related to inner enzymes, wall structure, stress, or other phenomenon that could have affected cell parameters. *Saccharomyces cerevisiae* (yeast) cells from the RS-16 strain were used. With the purpose of keeping the cell population in an identical physiological state, cells were grown in an aqueous medium with 1% yeast extract, 2% peptone, and 2% glucose at 28 °C in an agitator incubator at 200 rpm. The cells were gathered up after 48 hours at the end of the exponential growing phase, the cell growth was determined by absorbance at 660 nm. The cellular suspension was cleaned and re-suspended three times in 0.3 M mannitol by 2100 rpm centrifugation for 1 min. 

The concentration was measured by a Thoma chamber and adjusted to 5000 cells/mm^3^. At this concentration, maximum volume percent value is 0.075%. This value is close to the maximum of 0.1% that makes the assumption of dilute suspension valid. Moreover, the measurements were taken at the area where the cell velocity was high and the concentration was much less than this maximum. The suspension was left for one hour until the cells acquired the laboratory temperature of 24 °C. 

To avoid Joule effect, as low-conductive solutions as possible were used. The medium conductivity of the suspension was determined using a Crison CDTM-523 (L’Hospitalet de Llobregat, Barcelona, Spain) conductimeter at 3.8 kHz and adjusted at 2.2 mS/m.

In our work we wanted to use only viable cells. To verify that cell viability was greater than 95%, the methylene blue staining procedure [53] was used. None of the preparations had to be discarded for not fulfilling this condition.

### 3.3. Cell Radius Measurement

In this experiment, the diameter of the yeast and its speed cannot be measured at the same time. The cell radius was measured directly by microscopy, as it is defined in another study [16]. An inverted microscope using a 40× objective and a 1.6× multiplier were used to paper print forty frames. A total of 120 cells were evaluated. A calibrated micro-slide (Euromex, microscopes-Holland) that divides 1 mm into 100 parts was printed to convert pixels to length units. The radii were measured by hand, obtaining the maximum and minimum radius with a resolution of 0.02 µm/mm. The given radius for the cell was the media of both.

### 3.4. Experimental Procedure

A little amount of cellular suspension inside the disconnected chamber sealed with vacuum grease was charged. Afterward, the chamber was positioned vertically in an optical microscope. The electrodes were connected to the generator’s exit with an amplitude (15 V) and frequency (0.03, 0.05, 0.075, 0.1, 0.4, and 1 MHz) previously selected. After a few minutes of permitting the falling of cellular lumps, the cellular movement was recorded on VHS video. One single frequency was recorded for each experiment, and each experiment lasted less than five minutes.

The single and coupled cells came from the same preparation and were measured in the same experiment. Each experiment was repeated thrice. Couple cell formation is created from two cells that gather by chance. As described in the theoretical model, the only measured cells were those whose trajectories followed the *Y*-axis. It was chance that made the coupled cells displace together. The polarized cells formed “pearl chains” randomly when their trajectories were near [54,55,56].

As described in [17], the particles’ motion was recorded with a 1/2” CCD type video camera at (SONY) SSC-C531 (Tokyo, Japan) model, with a sensitive area of 6.3 × 4.7 mm, corresponding to a resolution of 500 × 582 pixels. The microscope was focused on the middle *Z*-axis to eliminate the area close to the glass that encloses the chamber. Both a computer generated reticule, marked in 9.09 μm increments, and a frame counter were superimposed onto the recording. The recording speed was 50 frames per second. The visual area of the microscope was 30 divisions in length along the vertical direction, ranging along the 15th (*y* = 136.35 μm) to the 45th (*y* = 409.05 μm), in relation to the coordinate origin. The recorded experiments were analyzed using the frame-advance mode of the VHS magnetoscope. The recorded experiments were analyzed visually. The division number, being crossed by the cell, and the frame count were both annotated. One frame of this experiment is shown in Figure 1b.

## 4. Results 

### 4.1. Measurement of the Electric Properties of the Dielectrophoretic Chamber

The results obtained are shown in Table 2.

**Figure 2:**
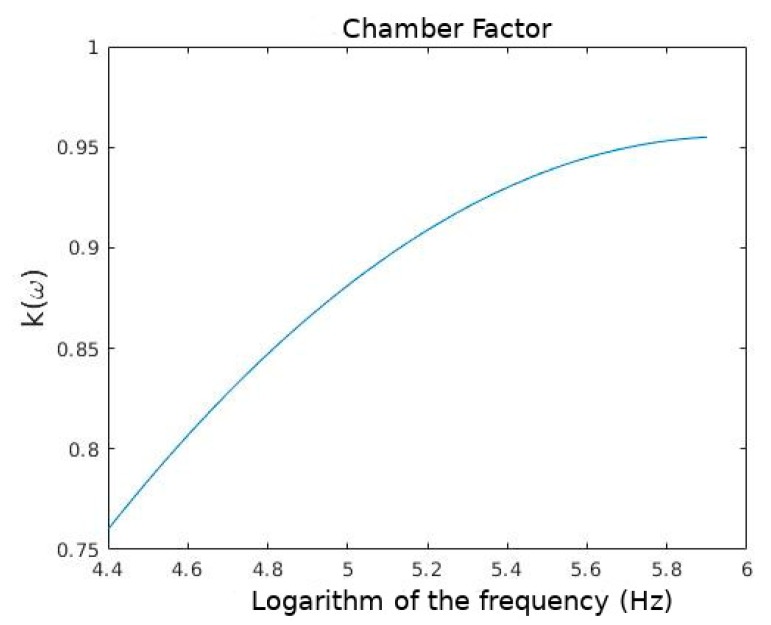
Frequency logarithm against *k*(*ω*).

The *k*(*ω*) values for Equation (9), as a frequency function, are shown in Figure 2:

### 4.2. Cell Radius Measurement

The population was characterized by an average value of 3.24 µm and a standard deviation of 0.4 µm.

### 4.3. Single Yeast Cells

The experiment was repeated thrice, and the chamber was dismantled, cleaned, and its electrodes were positioned in each preparation to check the repeatability of the experiment.

**Figure 3:**
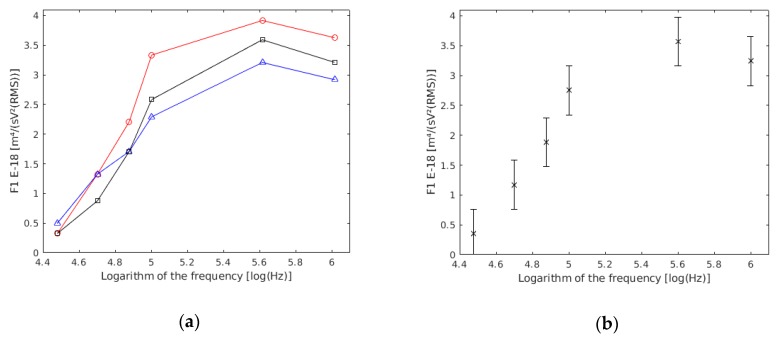
(**a**) $F_{1}$ results based on frequency obtained in each of the experiments conducted with single yeast cells. (**b**) Results of the three experiments together.

Through an ANOVA analysis, it was revealed that there were no statistically significant differences (*p* value >> 0.05) among the three experiments (Figure 3a). Therefore, the three experiments were considered as one (Figure 3b). In this way, more reliable data referring to each frequency was obtained. 

As described in the theoretical section, cells must follow the model of Equation (10). The difference in the absolute result of the individual regression of each cell may be due to three causes:

1. The difference between experiments was due to different solutions, camera mounting, etc.
2. Cells in a culture were not all the same (e.g., cells dying, metabolic changes, diameter, etc.).
3. The measured cell was different from the ideal theoretical model. For example, too many cells at the time of measurement, too many pearl chains on the electrodes which distorts the electric field in a point, accidental and occasional thermal or mechanical movements, error in the recording of manual data collection, etc.

The first point was discussed in this section. The second might also provide a good source of further research into the changing dielectric properties of cells that will be studied in other experiments.

To avoid the third circumstance, the methods from other studies were followed [16,17]. From the total of 442 cells measured, 273 cells were selected for the results because they had a coefficient of linear regression higher than 0.98, with the six measured points of velocity against Y^3^ used in the linear regression of Equation (10), and a sedimentation velocity located between −3 μm/s and 3 μm/s. With these requirements, 62% of the obtained data were used. The values for the $F_{1}$ parameter and the sedimentation velocity were obtained for each of the 273 selected cells. The statistical analysis of the sedimentation velocity ($v_{s}$) data indicates (*p* value >> 0.05) that $v_{s}$ is not significantly different from zero, which means it is negligible compared with the other measured velocities. For each of the 273 conducted regressions, the $F_{1}$ parameter was obtained with a value of the linear regression coefficient higher than 0.98, a null *p* value and a standard error lower than 5%. The obtained data are in Table 3.

### 4.4. Coupled Yeast Cells

Owing to the fact that the preparation was the same, the experiment was repeated thrice, exactly as it was in the single cells section. 

**Figure 4:**
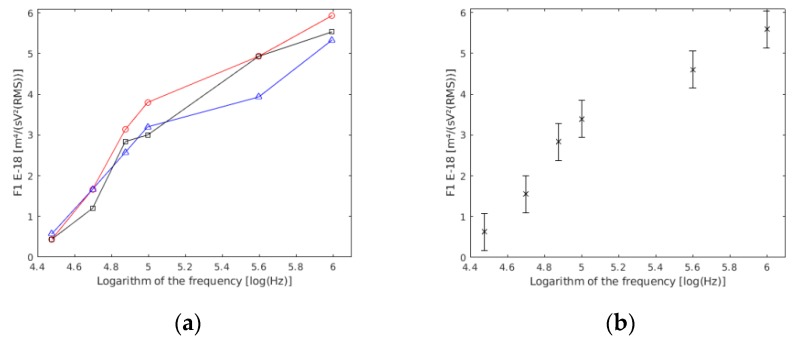
(**a**) $F_{1}$ results based on the frequency obtained in each of the experiences conducted with double yeast cells. $F_{1}$ units are m^4^/(sV^2^(RMS)). (**b**) Results of the three experiments together.

The statistical analysis of the ANOVA showed that there were no statistically significant differences between the three experiments (*p* value >> 0.05) (Figure 4a). This may be because of the variations in sample preparation, conductivity preparation, or the electrodes’ position, among others possibilities. However, these differences were not statistically relevant. Therefore, the three experiments were considered as one (Figure 4b). In this way, more reliable data referring to each frequency was obtained. 

The obtained data are shown in Table 4.

The values in Table 4 above are shown with a confidence interval of 99% in Figure 4b.

A total of 403 cells were measured. As outlined in other studies [16,17], 316 cells were selected for the results because they had a coefficient of linear regression higher than 0.98, with the six measured points used in the linear regression, and a sedimentation velocity located between −3 μm/s and 3 μm/s. With these requirements, 78% of the measured cells were used. The values for $F_{1}$ and $v_{s}$ were obtained for each couple. The statistical analysis revealed that $v_{s}$ was negligible (*p* value >> 0.05) in comparison with the rest of the measured velocities. For each selected couple, the $F_{1}$ parameter was obtained with a linear regression coefficient value higher than 0.98, and a null *p* value and a standard error lower than 5%. 

## 5. Discussion

### 5.1. Single Yeast Cells

To compare the experimental data of $F_{1}$ (Table 1) with bibliographic data, two articles were used. In the first of them [55], the dielectric spectroscopy method was used and two different cell models were proposed. In one of them, the cytoplasm was surrounded by a cellular membrane and a cellular wall, and in the second one, a vacuole compartment was added beside the above mentioned compartments. In the second article [57], the electrorotation technique and the three compartment cell model were used. 

**Figure 5:**
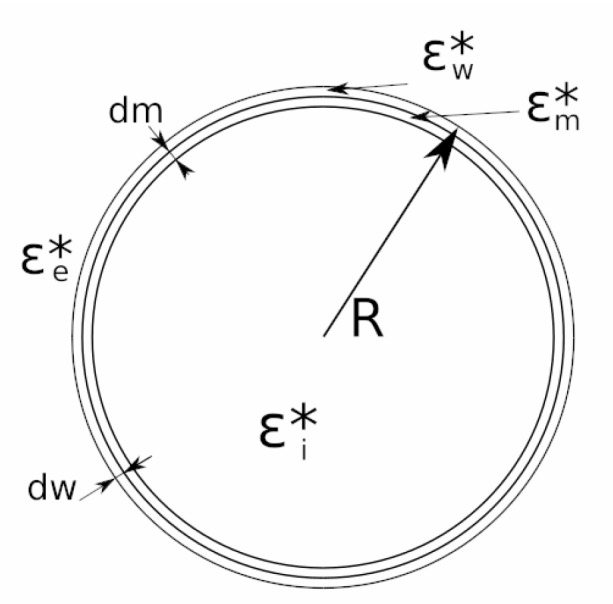
Yeast cell model with three compartments. ε_w_^*^: complex permittivity of the cell wall. ε_m_^*^: complex permittivity of the cell membrane. ε_e_^*^: extracellular medium permittivity. ε_i_^*^: intracellular medium permittivity. *dm:* cell membrane width. *dw:* cell wall width. *R:* cell radius.

In this work, the three compartment cell model has been considered. The model in which the vacuole compartment is taken into account has not been used because the vacuole compartment does not produce significant changes in the $F_{1}$ parameter until the range of GHz [58]. Because the measures were taken in the range of MHz, it was taken into account that the vacuole alterations are negligible. In Figure 5, the cellular model of three compartments is represented.

The results of the mentioned authors are shown in Table 5.

**Figure 6:**
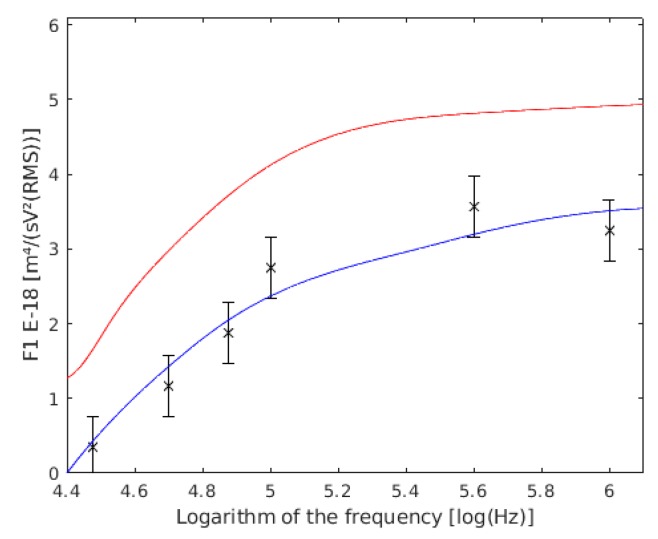
Comparison between our experimental data of $F_{1}$ and the values obtained by other authors, both of single cells. $F_{1}$ with the data of Holzel are shown in red, the data of Asami are shown in blue, and the results of this work are the six points with their confidence intervals drawn.

As can be assumed, the data of the membrane permittivity, wall conductivity, and wall thickness were different. The authors [57] affirmed that it was possible that they presented a 50% error rate in their measures, because of the membrane permittivity estimation. This error is related to the slight asymmetry of the positive peak of rotation. The discrepancy of this author for the wall conductivity was justified by the use of a different yeast strain. However, other authors with other strains and using the electrorotation method have found similar conductivities to those found using dielectric spectroscopy [56]. If this data is substituted into the $F_{1}$ expression for single cells, it is observed that our data (Figure 6) were closer to the Asami model [56] than to the Hölzel model [57].

To study how this model reacts to variations in its parameters, we have studied the model’s response to changes in the following parameters. 

#### 5.1.1. Cell Radius

**Figure 7:**
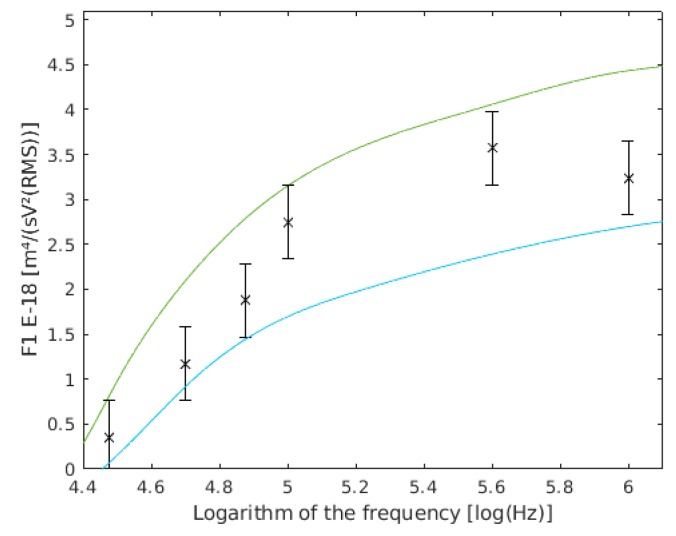
$F_{1}$ variation with the cell radius. $F_{1}$ with a 3.6 µm cell radius are shown in green, and with a 2.9 µm cell radius are shown in cyan, and the results of this work are the six frequency points with their confidence intervals drawn.

In our theoretical model, $F_{1}$ is very sensitive to cell radius variations. Our experimental results (Figure 7) are shown compared with those obtained directly from the theoretical model supposed to have a 10% variation in the cell radius. 

#### 5.1.2. Cell Membrane and Wall Thickness

**Figure 8:**
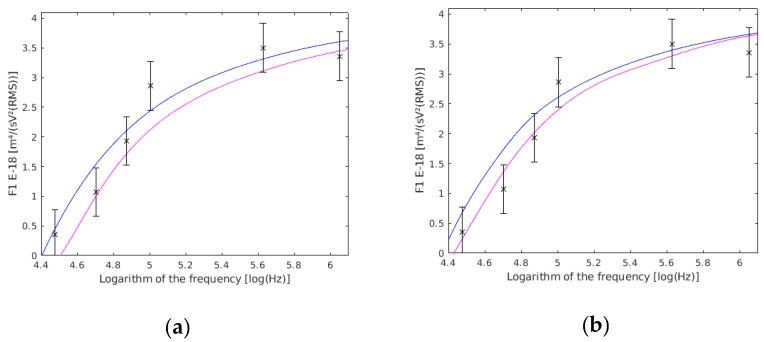
(**a**) $F_{1}$ variation with the cell wall thickness (dw). $F_{1}$ with a 0.275 µm cell wall thickness are shown in magenta, those with a 0.225 µm cell wall thickness are shown in blue, and the results of this work are the six frequency points with their confidence interval drawn. (**b**) $F_{1}$ variation with the cell membrane thickness (dm). $F_{1}$ with a 0.0077 µm cell membrane thickness are shown in magenta, those with a 0.0063 µm cell membrane thickness are shown in blue, and the results of this work are the six frequency points with their confidence intervals drawn.

The value of $F_{1}$ is almost the same against variations in the thickness of the wall (Figure 8a) and membrane (Figure 8b). This is because both thicknesses influence the real part of the Clausius–Mossoti factor and could only be determined at the same time. Nevertheless, the cell squared radius directly influences the value of $F_{1}$. Besides, for the inner part of the compartment, its influence will be lower while working with lower frequencies (as it has been used in this work), and greater at higher frequencies.

#### 5.1.3. Cell Wall Conductivity and Permittivity

**Figure 9:**
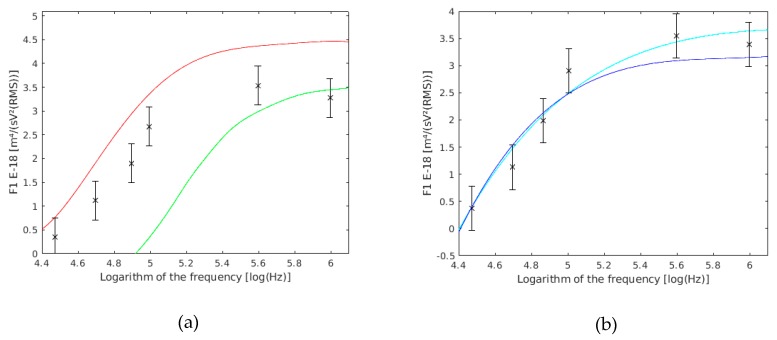
(**a**) $F_{1}$ variation with the cell wall conductivity (σ_p_). $F_{1}$ with a 11 mS/m cell wall conductivity are shown in red, those with a 0.11 mS/m cell wall conductivity are shown in green, and the results of this work are the six frequency points with their confidence intervals drawn. (**b**) $F_{1}$ variation with the cell wall relative permittivity (ε_ω_). $F_{1}$ with an 80 cell wall relative permittivity are shown in cyan, those with a 40 cell wall relative permittivity are shown in blue, and the results of this work are the six frequency points with their confidence intervals drawn.

The cell wall conductivity is one of the most influential parameters over $F_{1}$ (Figure 9a). Because the cell wall is made of mucopolysaccharides, its conductivity and permittivity (Figure 9b) are the function of the medium in which they are immersed. Some authors [56] take the cell wall conductivity as proportional to the external medium conductivity. This is only valid for cells in perfect conditions. In the case where these cells had their membrane damaged, this conductivity could be proportional to the cytoplasm conductivity, which is usually greater in dielectrophoresis experiments.

#### 5.1.4. Cell Membrane Relative Permittivity and Conductivity

**Figure 10:**
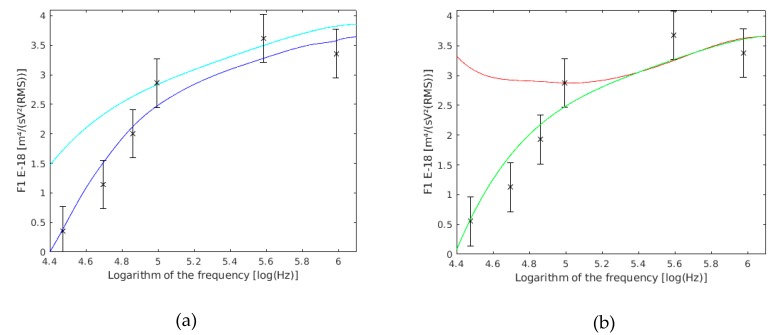
(**a**) $F_{1}$ variation with the cell membrane relative permittivity (ε_m_). $F_{1}$ with 10.4 cell membrane relative permittivity are shown in cyan, those with a 5.2 cell membrane relative permittivity are shown in blue, and the results of this work are the six frequency points with their confidence intervals drawn. (**b**) $F_{1}$ variation with the cell membrane conductivity (σ_m_). $F_{1}$ with 0.001 mS/m cell membrane conductivity are shown in red, those with 0.01 mS/m cell wall conductivity are shown in green, and the results of this work are the six frequency points with their confidence interval drawn.

The slight changes in membrane permittivity cause important variations in $F_{1}$ (Figure 10a). No reason was found in bibliography for the change in cell membrane permittivity. It is commonly placed in a range between 4 and 10, and is often used to adjust the experimental values to the theoretical model.

The cell membrane conductivity is usually so low that it does not even affect the measurements. Therefore, it can be considered zero. On the other hand, when a change in cell membrane conductivity from 10^−3^ to 10^−2^ S/m takes places, a change is produced in the theoretical factor behavior (Figure 10b). It means that if this conductivity is kept below 10^−2^ S/m, its variation will not at all influence the parameter measured. However, it will be bound to take this conductivity into account if it takes higher values. The conductivity value could rise owing to a membrane perforation, as a result of the action of external agents.

#### 5.1.5. Cell Cytoplasm Conductivity and Relative Permittivity

**Figure 11:**
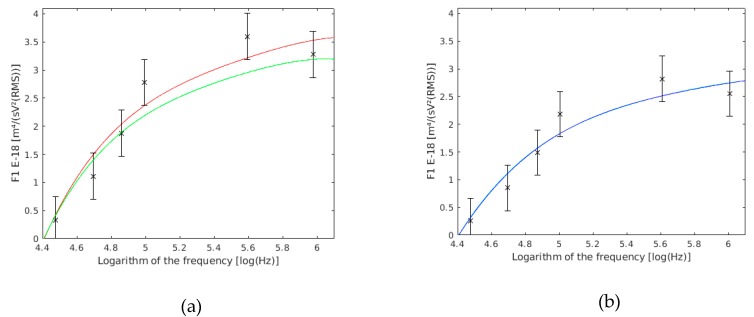
(**a**) $F_{1}$ variation with the cell cytoplasm conductivity (σ_i_). $F_{1}$ with 6 S/m inner cell conductivity are shown in red, those with 0.06 S/m cell wall conductivity are shown in green, and the results of this work are the six frequency points with their confidence intervals drawn. (**b**) $F_{1}$ variation with the cell cytoplasm relative permittivity (ε_i_) $F_{1}$. with 80 inner cell permittivity are shown in cyan, those with 40 inner cell permittivity are shown in blue, and the results of this work are the six frequency points with their confidence intervals drawn. It seems to be only one line because both lines are overlapped.

As it is revealed by Figure 11a,b, in the frequency range studied, variations in cell cytoplasm do not have a strong influence over $F_{1}$, as had the prior parameters. At higher frequencies, the cytoplasm properties become more important.

### 5.2. Coupled Yeast Cells

This cell formation is created from two cells that have gathered by chance, and the electric field action keeps them together afterwards by means of the dipole formation (pearl chain effect) [54,55,56]. In other words, at first, these cells are found together by chance, and afterwards, the electric field transforms each one of the cells that form these couples in a dipole. At the time when they are dipoles, the couple will be kept joined together by means of the electric force exerted over each other.

**Figure 12:**
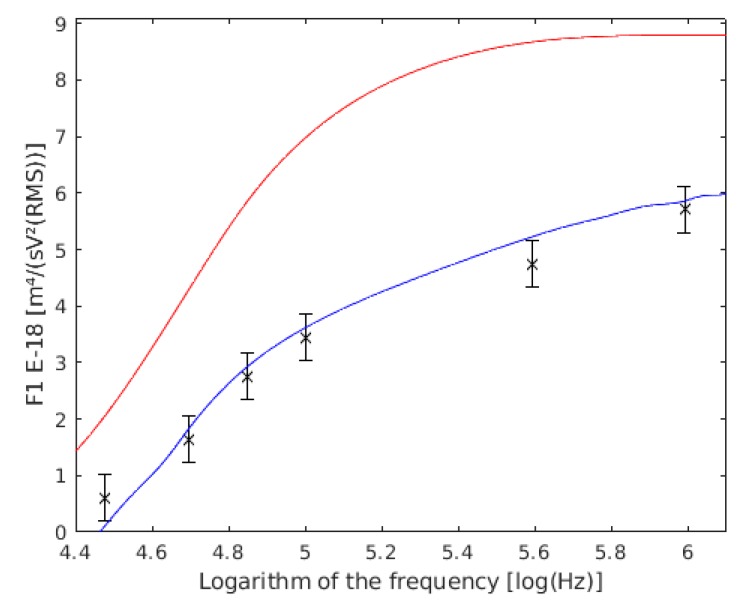
Comparison between our experimental data of $F_{1}$ for double cells and the values obtained by Asami (1996) [56] and Hölzel (1992) [57] models. $F_{1}$ with the data of Holzel are shown in red, the data of Asami are shown in blue, and the results of this work are the six points with their confidence intervals drawn.

The measurements are compared with the same authors’ study results from the single cells experiments (Figure 12).

## 6. Conclusions

The statistical analysis confirmed the theoretical model about the symmetry axis of the electric field, and showed that the used method is suitable and accurate for the dynamic measurement of the dielectrophoretic force in single and coupled cells.

The measures obtained were similar to the ones collected in a previous bibliography with the advantage that the setup can be dismantled and assembled again with high repeatability. The differences of $F_{1}$ obtained for coupled and single cell results can be appreciated. Likewise, because the experimental results for single and coupled cells respond to the same theoretical values of conductivity and permittivity for different compartments, the validity of the theoretical model in duplicate is confirmed. 

On the other hand, there are plenty of combinations of electric parameters that give a similar cell behavior. In other words, our method is not capable of measuring different parameters in an isolated way. What is possible is to obtain one of these parameters by setting the other ones. It is possible to discover whether a population has suffered a sudden change in one of these parameters (because of some external agent) or not. Likewise, the variation of electric potential has been checked as a technique to reach higher velocities to register the proper velocity for our measurement system. 

Additionally, the chamber has two limitations:

- Conductivity: it would be very useful to vary the solution conductivity so as to look for the membrane capacity. Nonetheless, it is not possible, because of the appearance of thermal currents by the time the conductivity is increased.
- Frequency: to obtain a more complete spectrum, the frequency was varied, and the following problems were observed:
  ∘ The higher the frequency, the lower the velocity—an effect which is not explained by electrode polarization or by dielectric properties of particles.
  ∘ The frequency was reduced to reach negative dielectrophoresis, when thermal currents appeared, making any negative dielectrophoresis visualization impossible.

These limitations made it impossible to measure the conductivity and permittivity values of different cellular compartments at the same time. Nevertheless, it was possible to calculate the values of one of these dielectric parameters by setting the rest and observing variations in cellular DEP behavior due to variations in any of them. The dielectric parameters of the yeast cells that most influence their DEP behavior are the cell diameter, the wall conductivity, and the conductivity and permittivity of the membrane. 

To verify the practical utility of this method, in future works we will measure *F*_1_ parameters for the same cell population in different physiological states. 

## Figures and Tables

**Table 1:** Mathematical expressions of $F_{1}$ and $v_{s}$.

	$F_{1}\;(m^{4}/({\mathrm{sV}}^{2}(\mathrm{RMS}))$	$v_{s}\;(m/s)$
Single cells	2R2εmRe[fCM]3ηα2	$\frac{2R^{2}\rho_{pm}g}{9\eta }$
Coupled cells	23R2εmRe[2fCM1−fCM4]1.44ηα2	$v_{s}=\frac{4R^{2}\rho_{mp}g}{1.44\eta 9}$

**Table 2:** Electric properties of the dielectrophoretic chamber.

Parallel Resistance (Ω)	18,500
Parallel Capacitance (F)	$1.2\times 10^{-13}$

**Table 3:** $F_{1}$ parameter measurements. Collected values for single cells.

Frequency (kHz)	$F_{1}10^{-18}$ $\left(\frac{m^{4}}{V^{2}\left(rms\right)S}\right)$	Standard Error $\times 10^{-18}$	Selected Cells	Measured Cells
30	0.35	0.4	8	42
50	1.17	0.1	36	61
75	1.88	0.1	52	72
100	2.75	0.1	48	69
400	3.57	0.1	57	95
1000	3.24	0.1	72	103

**Table 4:** Collected values for coupled cells.

Frequency (kHz)	$F_{1}\cdot 10^{-18}$ $\left(\frac{m^{4}}{V^{2}\left(rms\right)S}\right)$	Standard Error $\times 10^{-18}$	Selected Cells	Measured Cells
30	0.63	0.2	32	49
50	1.55	0.2	47	58
75	2.83	0.1	56	67
100	3.39	0.1	56	64
400	4.60	0.1	58	85
1000	5.59	0.1	67	80

**Table 5:** Comparison between the values obtained by Asami (1996) [56] and Hölzel (1992) [57].

	Asami and Yonezawa (1996) [56]	Hölzel and Lamprecht (1992) [57]
Conductivity (S/m)		
σ_e_ exterior	0.1	$2.5\times 10^{-3}$
σ_w_ wall	$0.2\sigma_{e}-0.24\sigma_{e}$	$1\times 10^{-2}-5\times 10^{-2}$
σ_m_ membrane	0	$\;2.5\times 10^{-8}$
σ_i_ inner	0.6	0.55
Relative permittivity (-)		
ε_e_ exterior	77	-
ε_w_ wall	60	-
ε_m_ membrane	5.2	7.6
ε_i_ inner	58	-
Geometric parameters (m)		
R–cell radius	$2.35\times 10^{-6}$	-
dw–wall thickness	$0.25\times 10^{-6}$	$0.11\times 10^{-6}$
dm–membrane thickness	$7\times 10^{-9}$	-
